# Supplementary figures and images for: Loss of ZNRF3/RNF43 unleashes EGFR in cancer (part 1 of 2)
Source: eLife. 2026 Apr 10;13:RP95639. doi: 10.7554/eLife.95639 (PMC13068435; doi:10.7554/eLife.95639)

Figure 2B

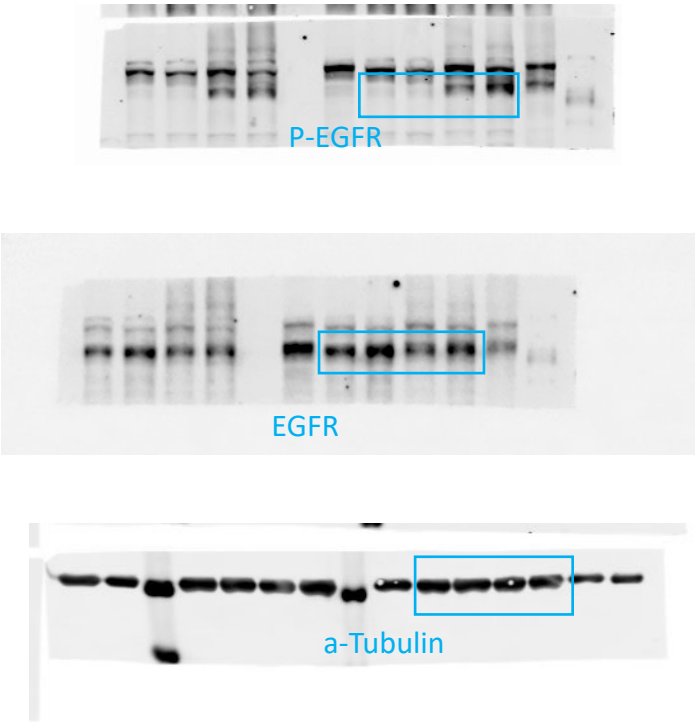

Supplement: Figure 2—source data 2. [file elife-95639-fig2-data2.zip › Figure 2B.pdf]

Figure 2C

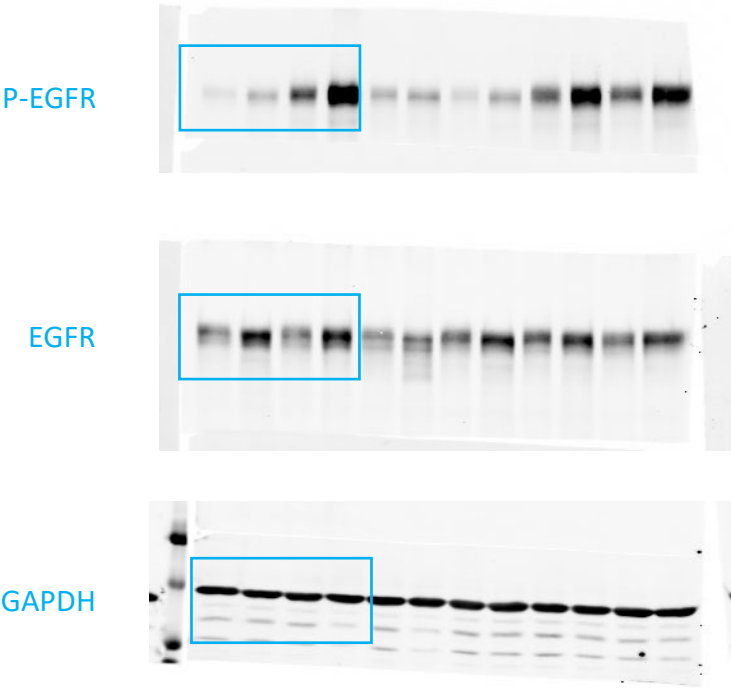

Supplement: Figure 2—source data 2. [file elife-95639-fig2-data2.zip › Figure 2C.pdf]

Figure 2D

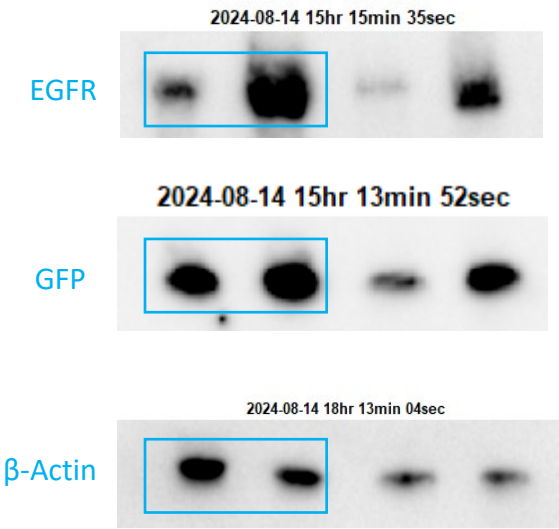

Supplement: Figure 2—source data 2. [file elife-95639-fig2-data2.zip › Figure 2D.pdf]

Figure 2E

HT-29

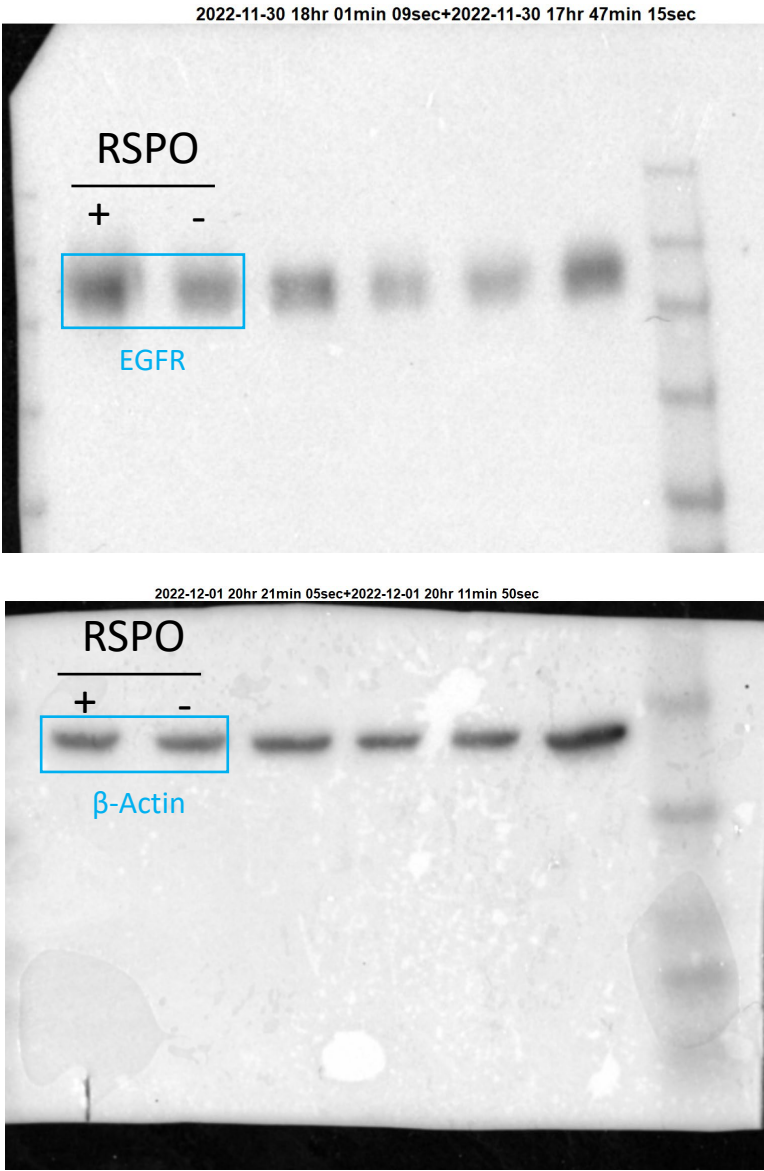

Supplement: Figure 2—source data 2. [file elife-95639-fig2-data2.zip › Figure 2E.pdf]

Figure 2F

EGFR

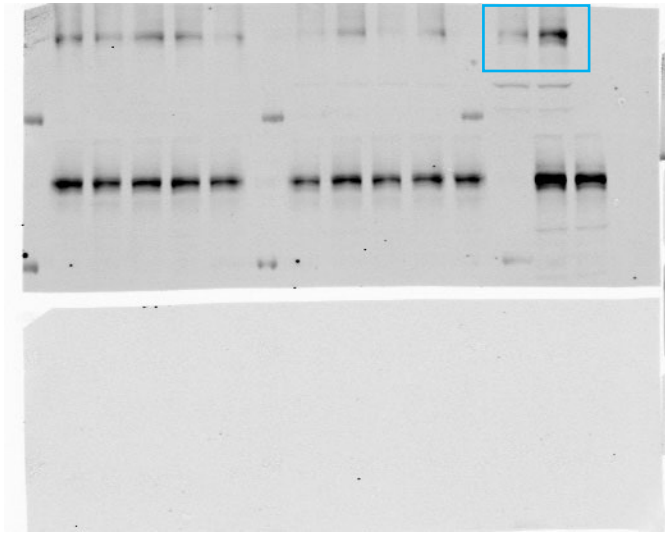

GAPDH

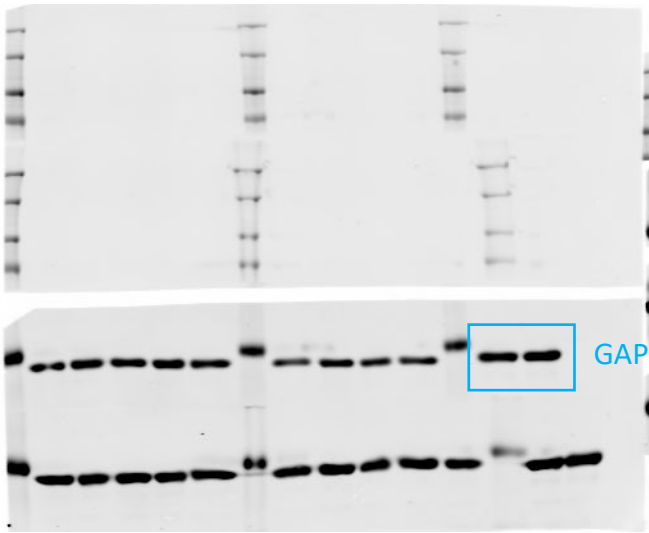

Supplement: Figure 2—source data 2. [file elife-95639-fig2-data2.zip › Figure 2F.pdf]

Figure 2G

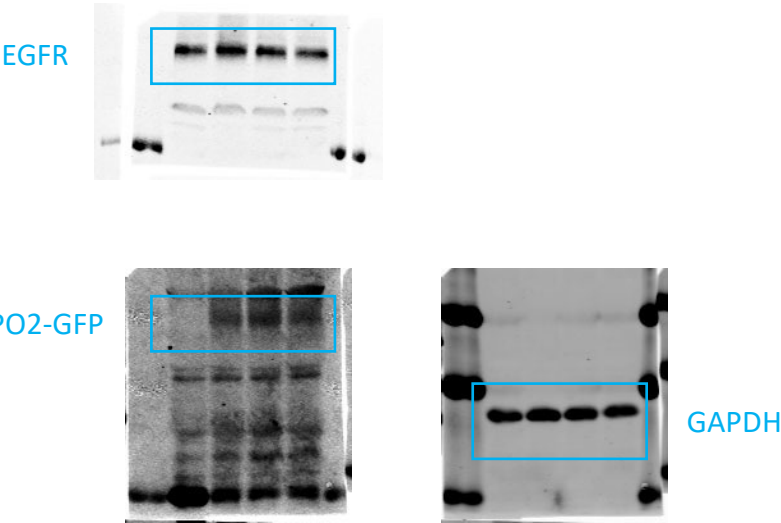

Supplement: Figure 2—source data 2. [file elife-95639-fig2-data2.zip › Figure 2G.pdf]

Figure 2A

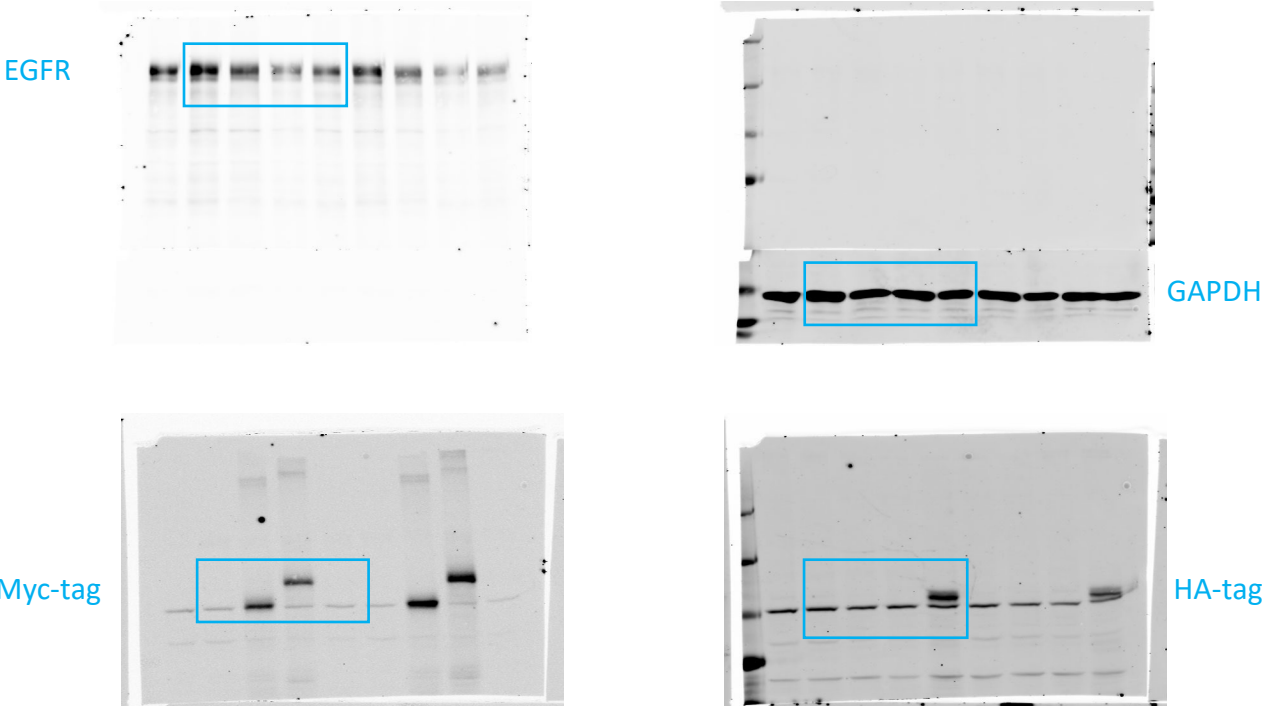

Supplement: Figure 2—source data 2. [file elife-95639-fig2-data2.zip › Figure 2A.pdf]

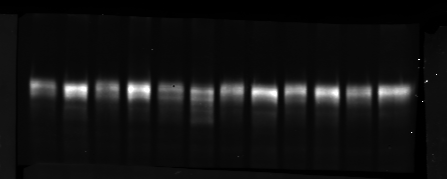

Supplement: Figure 2—source data 3. [file elife-95639-fig2-data3.zip › Figure 2C - EGFR_800.tif]

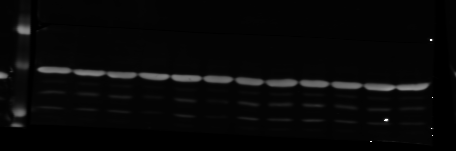

Supplement: Figure 2—source data 3. [file elife-95639-fig2-data3.zip › Figure 2C - GAPDH_700.tif]

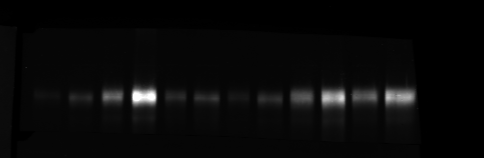

Supplement: Figure 2—source data 3. [file elife-95639-fig2-data3.zip › Figure 2C - pEGFR_800.tif]

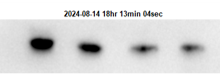

Supplement: Figure 2—source data 3. [file elife-95639-fig2-data3.zip › Figure 2D - bActin.png]

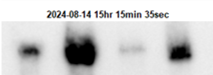

Supplement: Figure 2—source data 3. [file elife-95639-fig2-data3.zip › Figure 2D - EGFR.tif]

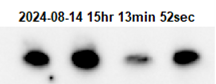

Supplement: Figure 2—source data 3. [file elife-95639-fig2-data3.zip › Figure 2D - GFP.tif]

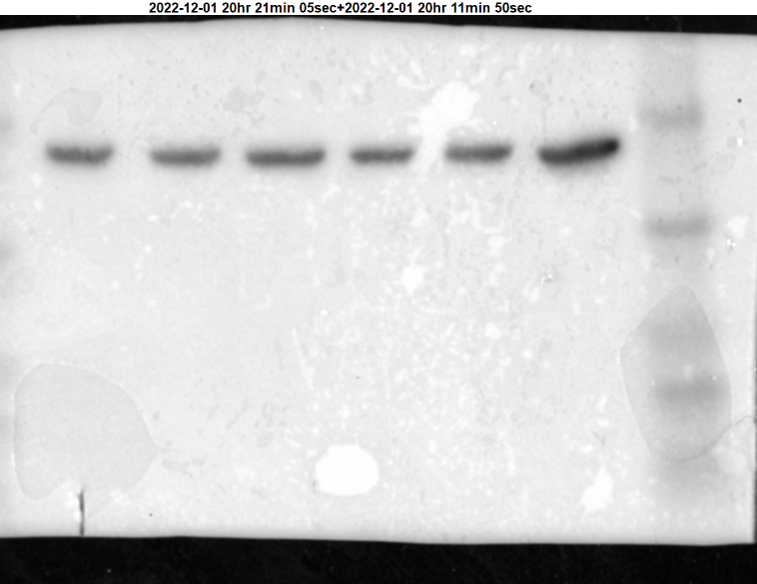

Supplement: Figure 2—source data 3. [file elife-95639-fig2-data3.zip › Figure 2E - bactin.tif]

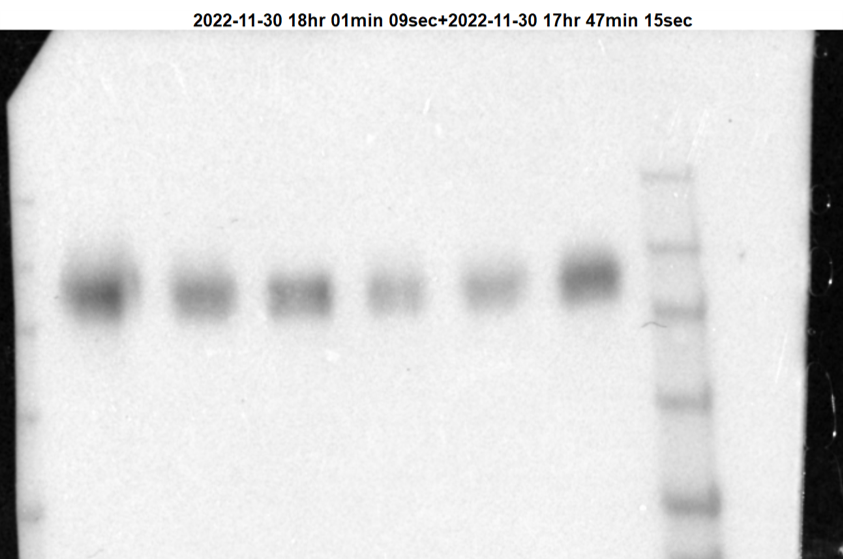

Supplement: Figure 2—source data 3. [file elife-95639-fig2-data3.zip › Figure 2E - EGFR.tif]

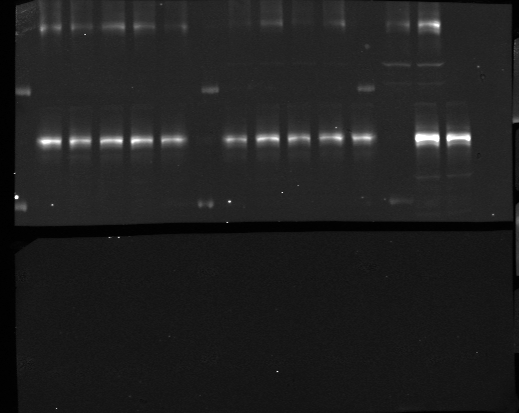

Supplement: Figure 2—source data 3. [file elife-95639-fig2-data3.zip › Figure 2F - EGFR_800.tif]

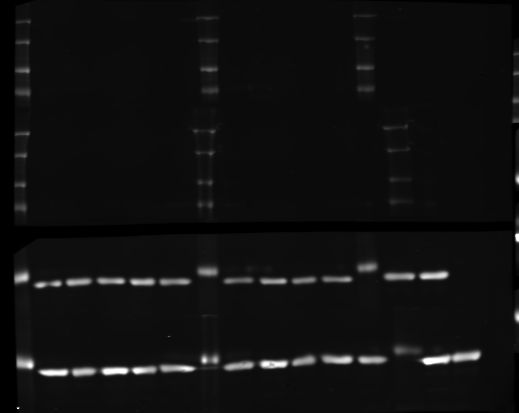

Supplement: Figure 2—source data 3. [file elife-95639-fig2-data3.zip › Figure 2F - GAPDH_700.tif]

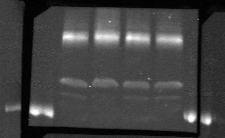

Supplement: Figure 2—source data 3. [file elife-95639-fig2-data3.zip › Figure 2G - EGFR_800.tif]

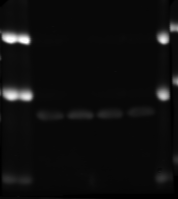

Supplement: Figure 2—source data 3. [file elife-95639-fig2-data3.zip › Figure 2G - GAPDH_700.tif]

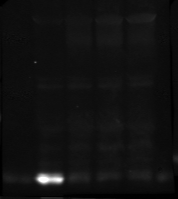

Supplement: Figure 2—source data 3. [file elife-95639-fig2-data3.zip › Figure 2G - RSPO-GFP_800.tif]

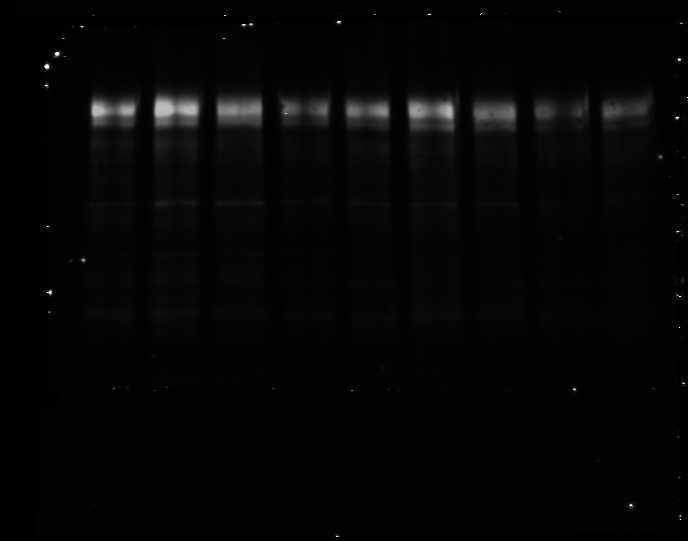

Supplement: Figure 2—source data 3. [file elife-95639-fig2-data3.zip › Figure 2A - EGFR_800.tif]

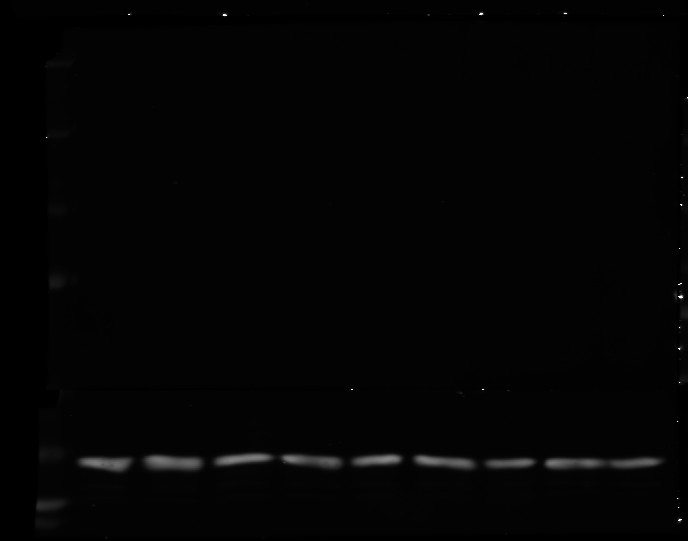

Supplement: Figure 2—source data 3. [file elife-95639-fig2-data3.zip › Figure 2A - GAPDH_700.tif]

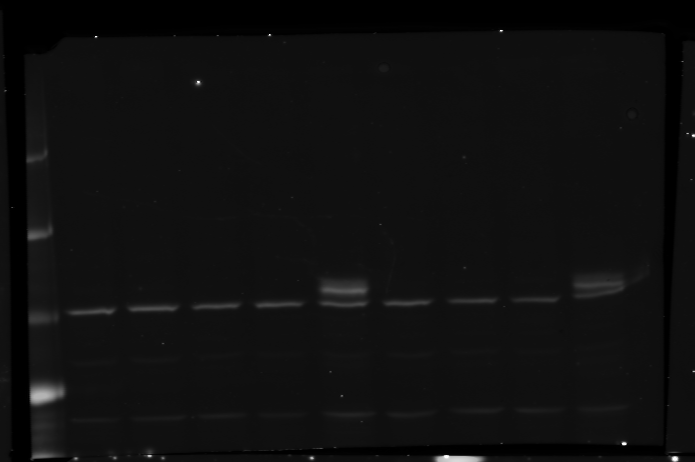

Supplement: Figure 2—source data 3. [file elife-95639-fig2-data3.zip › Figure 2A - HA-tag_700.tif]

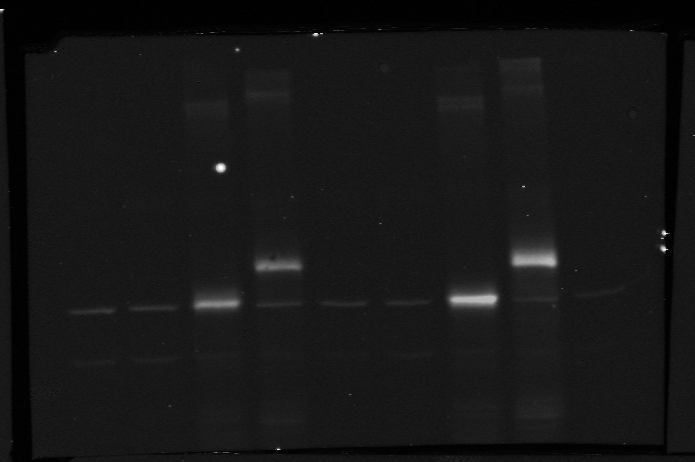

Supplement: Figure 2—source data 3. [file elife-95639-fig2-data3.zip › Figure 2A - Myc-tag_800.tif]

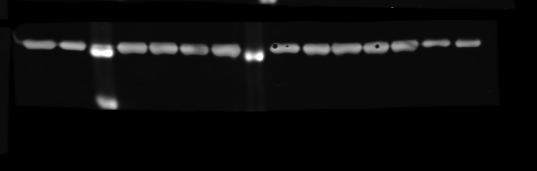

Supplement: Figure 2—source data 3. [file elife-95639-fig2-data3.zip › Figure 2B - aTubulin_700.tif]

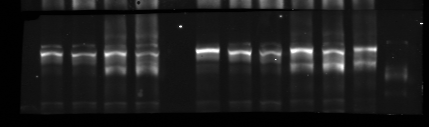

Supplement: Figure 2—source data 3. [file elife-95639-fig2-data3.zip › Figure 2B - pEGFR_800.tif]

Figure 2 - Figure supplement 1E

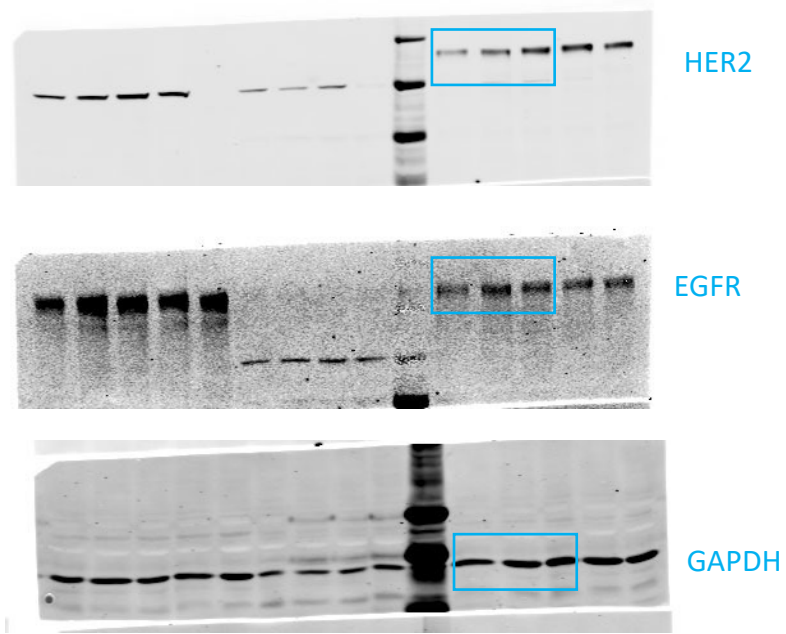

Supplement: Figure 2—figure supplement 1—source data 2. [file elife-95639-fig2-figsupp1-data2.zip › Figure 2 - Figure supplement 1E.pdf]

Figure 2 - Figure supplement 1F

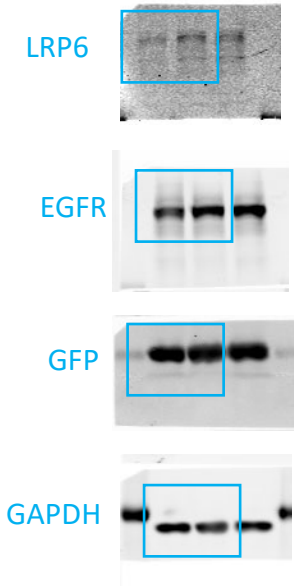

Supplement: Figure 2—figure supplement 1—source data 2. [file elife-95639-fig2-figsupp1-data2.zip › Figure 2 - Figure supplement 1F.pdf]

Figure 2 - Figure supplement 1G

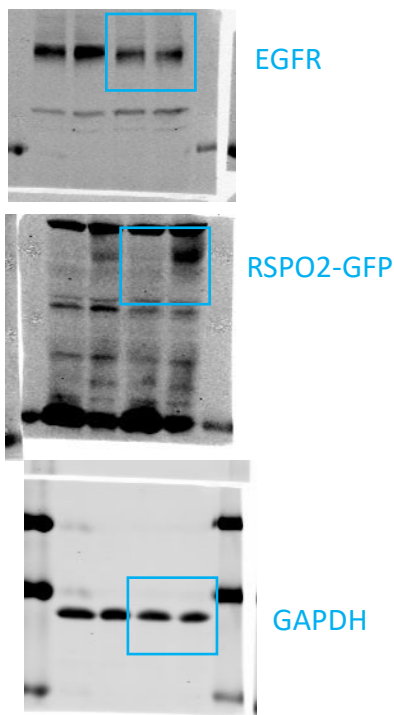

Supplement: Figure 2—figure supplement 1—source data 2. [file elife-95639-fig2-figsupp1-data2.zip › Figure 2 - Figure supplement 1G.pdf]

Figure 2 - Figure supplement 1A

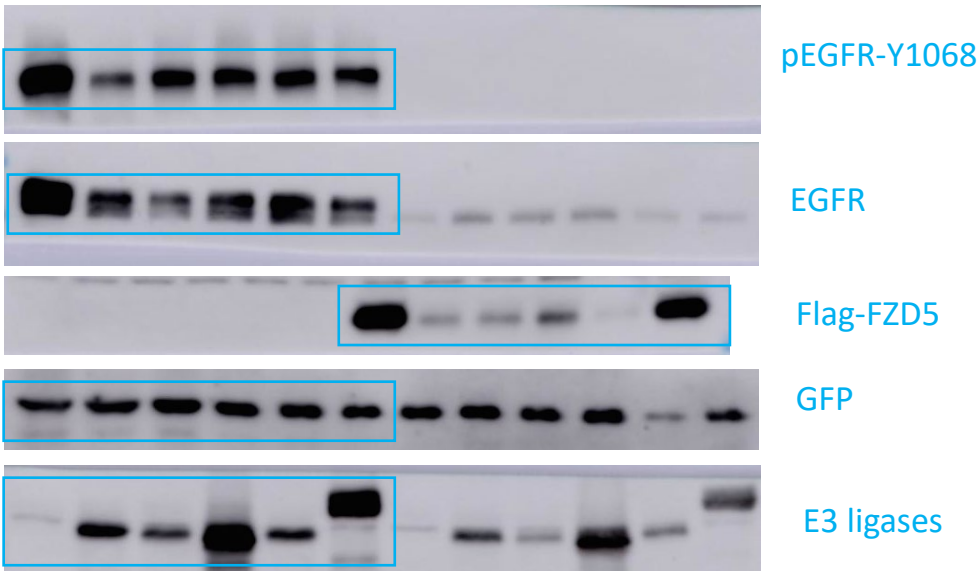

Supplement: Figure 2—figure supplement 1—source data 2. [file elife-95639-fig2-figsupp1-data2.zip › Figure 2 - Figure supplement 1A.pdf]

Figure 2 - Figure supplement 1B

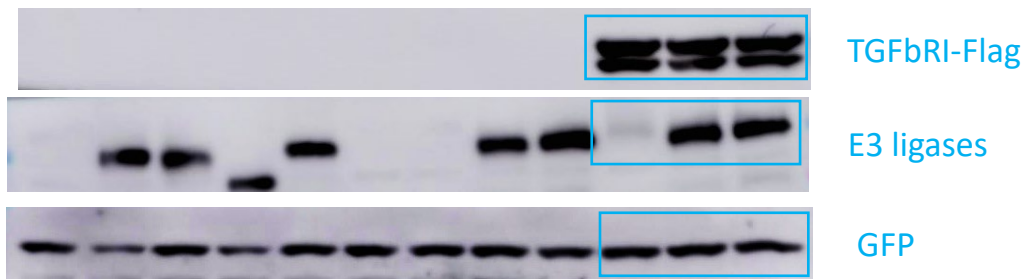

Supplement: Figure 2—figure supplement 1—source data 2. [file elife-95639-fig2-figsupp1-data2.zip › Figure 2 - Figure supplement 1B.pdf]

Figure 2 - Figure supplement 1C

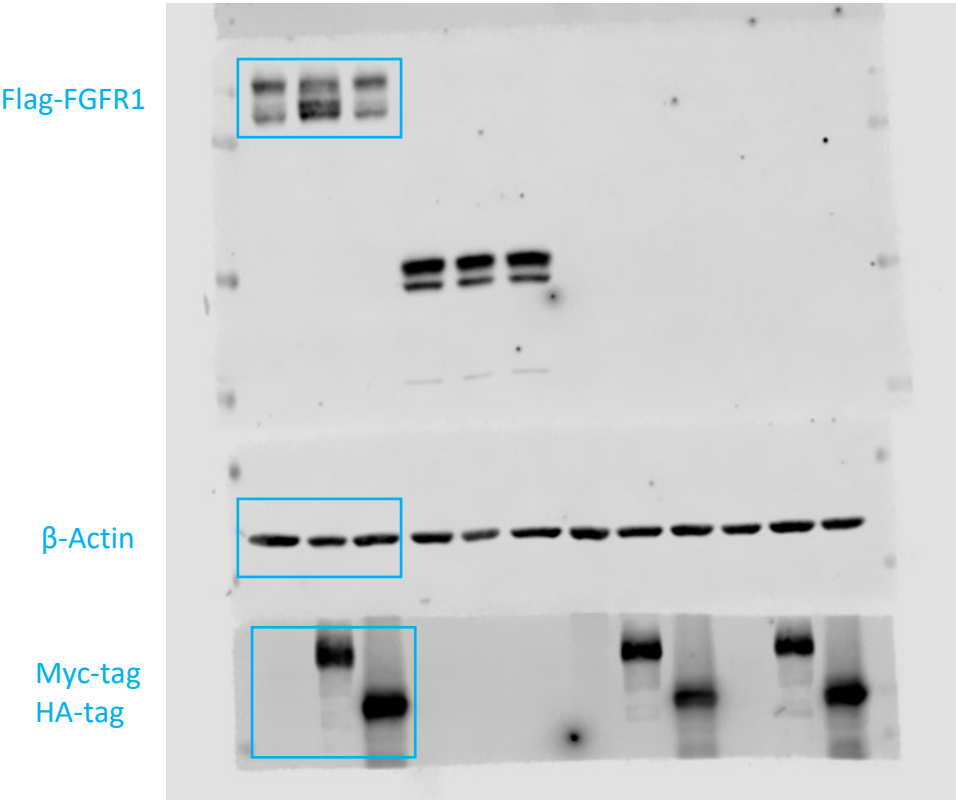

Supplement: Figure 2—figure supplement 1—source data 2. [file elife-95639-fig2-figsupp1-data2.zip › Figure 2 - Figure supplement 1C.pdf]

Figure 2 - Figure supplement 1D

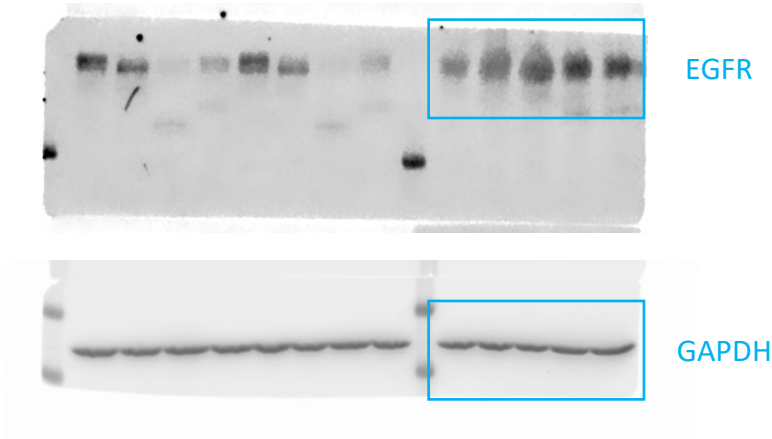

Supplement: Figure 2—figure supplement 1—source data 2. [file elife-95639-fig2-figsupp1-data2.zip › Figure 2 - Figure supplement 1D.pdf]

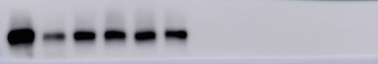

Supplement: Figure 2—figure supplement 1—source data 3. [file elife-95639-fig2-figsupp1-data3.zip › Fig2S1A - pEGFR-Y1068.tif]

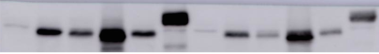

Supplement: Figure 2—figure supplement 1—source data 3. [file elife-95639-fig2-figsupp1-data3.zip › Fig2S1A- E3 ligases.tif]

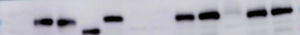

Supplement: Figure 2—figure supplement 1—source data 3. [file elife-95639-fig2-figsupp1-data3.zip › Fig2S1B - E3 Ligases.tif]

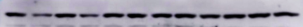

Supplement: Figure 2—figure supplement 1—source data 3. [file elife-95639-fig2-figsupp1-data3.zip › Fig2S1B - GFP.tif]

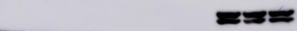

Supplement: Figure 2—figure supplement 1—source data 3. [file elife-95639-fig2-figsupp1-data3.zip › Fig2S1B - TGFbRI-Flag.tif]

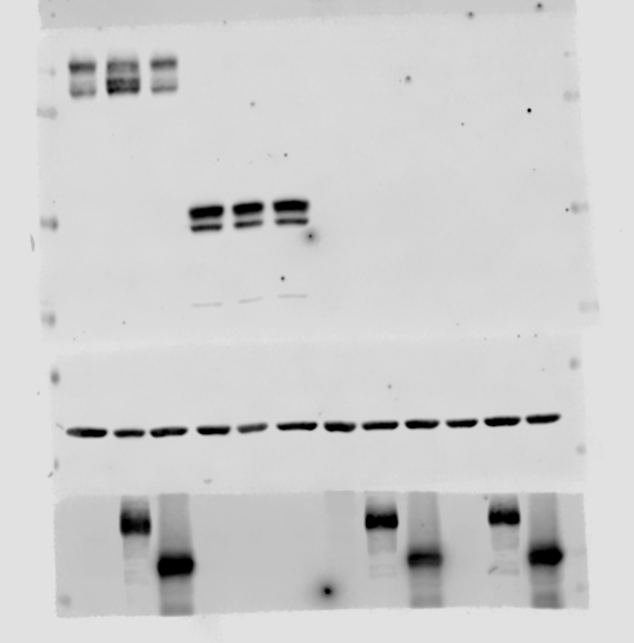

Supplement: Figure 2—figure supplement 1—source data 3. [file elife-95639-fig2-figsupp1-data3.zip › Fig2S1C - FGFR_E3ligase.tif]

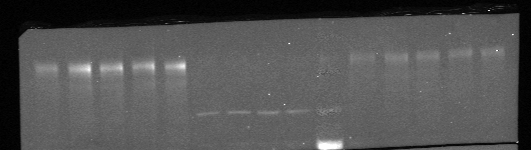

Supplement: Figure 2—figure supplement 1—source data 3. [file elife-95639-fig2-figsupp1-data3.zip › Fig2S1E - EGFR_800.tif]

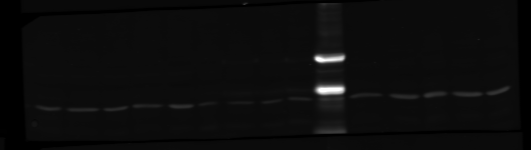

Supplement: Figure 2—figure supplement 1—source data 3. [file elife-95639-fig2-figsupp1-data3.zip › Fig2S1E - GAPDH_700.tif]

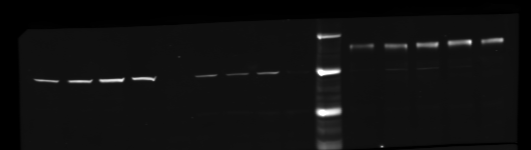

Supplement: Figure 2—figure supplement 1—source data 3. [file elife-95639-fig2-figsupp1-data3.zip › Fig2S1E - HER2_700.tif]

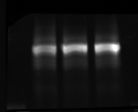

Supplement: Figure 2—figure supplement 1—source data 3. [file elife-95639-fig2-figsupp1-data3.zip › Fig2S1F - EGFR_800.tif]

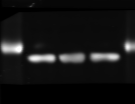

Supplement: Figure 2—figure supplement 1—source data 3. [file elife-95639-fig2-figsupp1-data3.zip › Fig2S1F - GAPDH_700.tif]

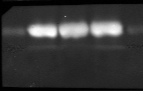

Supplement: Figure 2—figure supplement 1—source data 3. [file elife-95639-fig2-figsupp1-data3.zip › Fig2S1F - GFP_800.tif]

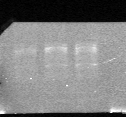

Supplement: Figure 2—figure supplement 1—source data 3. [file elife-95639-fig2-figsupp1-data3.zip › Fig2S1F - LRP6_800.tif]

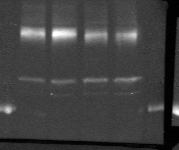

Supplement: Figure 2—figure supplement 1—source data 3. [file elife-95639-fig2-figsupp1-data3.zip › Fig2S1G - EGFR-800.tif]

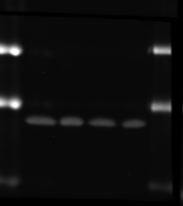

Supplement: Figure 2—figure supplement 1—source data 3. [file elife-95639-fig2-figsupp1-data3.zip › Fig2S1G - GAPDH-700.tif]

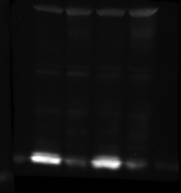

Supplement: Figure 2—figure supplement 1—source data 3. [file elife-95639-fig2-figsupp1-data3.zip › Fig2S1G - GFP-800.tif]

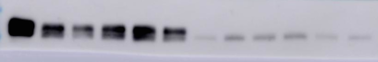

Supplement: Figure 2—figure supplement 1—source data 3. [file elife-95639-fig2-figsupp1-data3.zip › Fig2S1A - EGFR.tif]

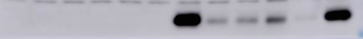

Supplement: Figure 2—figure supplement 1—source data 3. [file elife-95639-fig2-figsupp1-data3.zip › Fig2S1A - Flag-FZD5.tif]

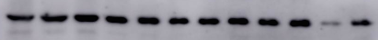

Supplement: Figure 2—figure supplement 1—source data 3. [file elife-95639-fig2-figsupp1-data3.zip › Fig2S1A - GFP.tif]

Figure 3C

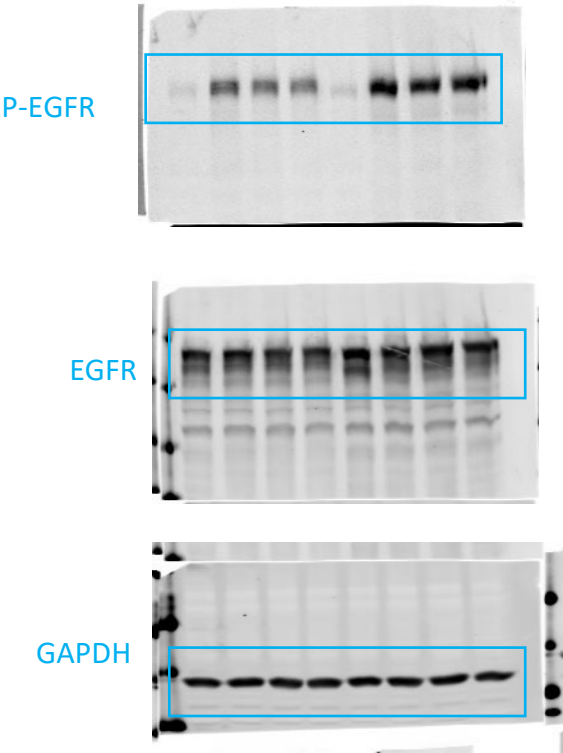

Supplement: Figure 3—source data 2. [file elife-95639-fig3-data2.zip › Figure_3_source_data_2.pdf]

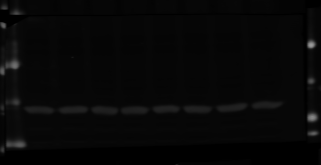

Supplement: Figure 3—source data 3. [file elife-95639-fig3-data3.zip › Figure 3c - GAPDH_700.tif]

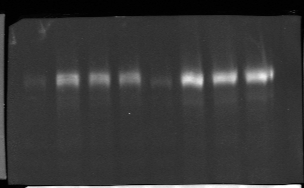

Supplement: Figure 3—source data 3. [file elife-95639-fig3-data3.zip › Figure 3c - pEGFR_800.tif]

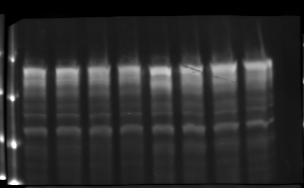

Supplement: Figure 3—source data 3. [file elife-95639-fig3-data3.zip › Figure 3c - EGFR_700.tif]

Figure 4A

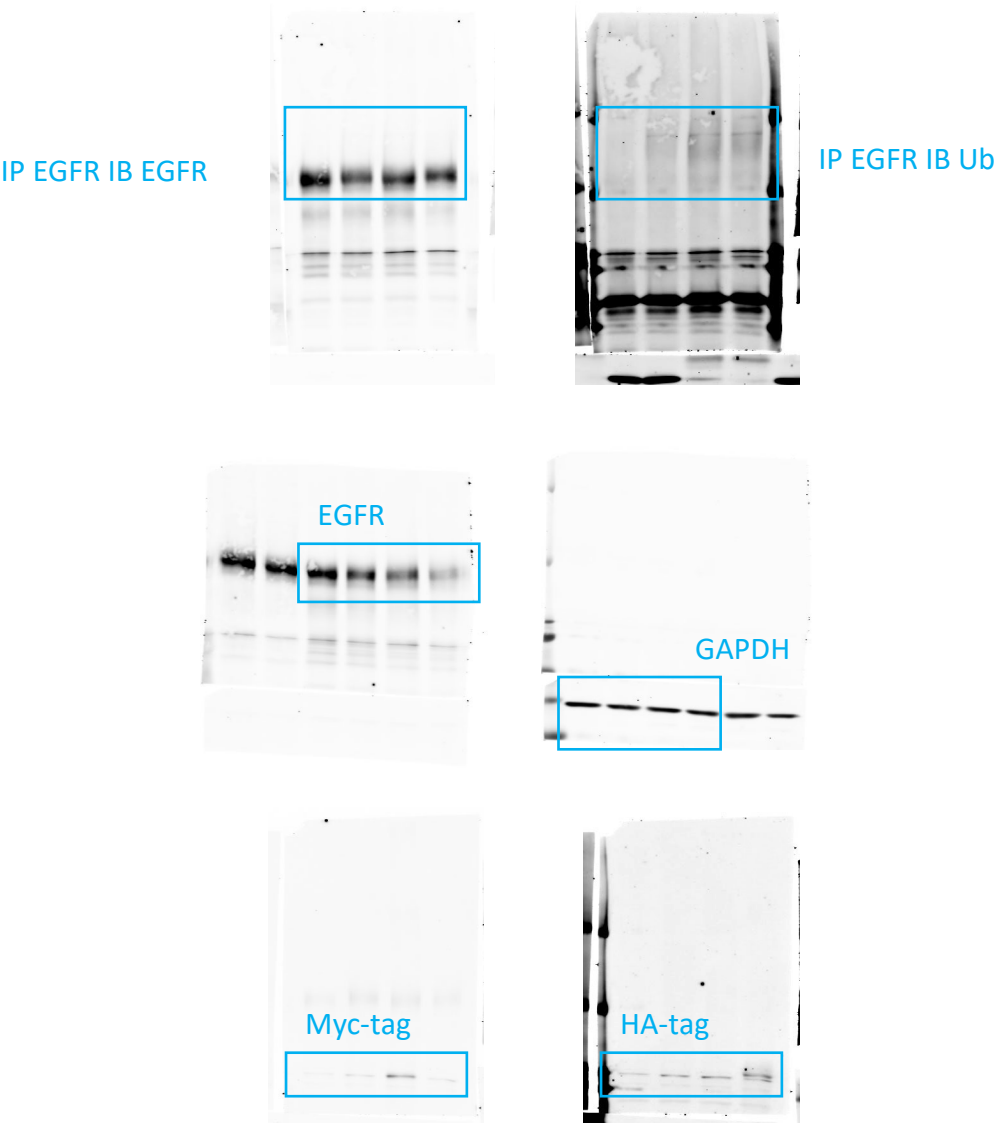

Supplement: Figure 4—source data 1. [file elife-95639-fig4-data1.zip › Figure 4A.pdf]

Figure 4B

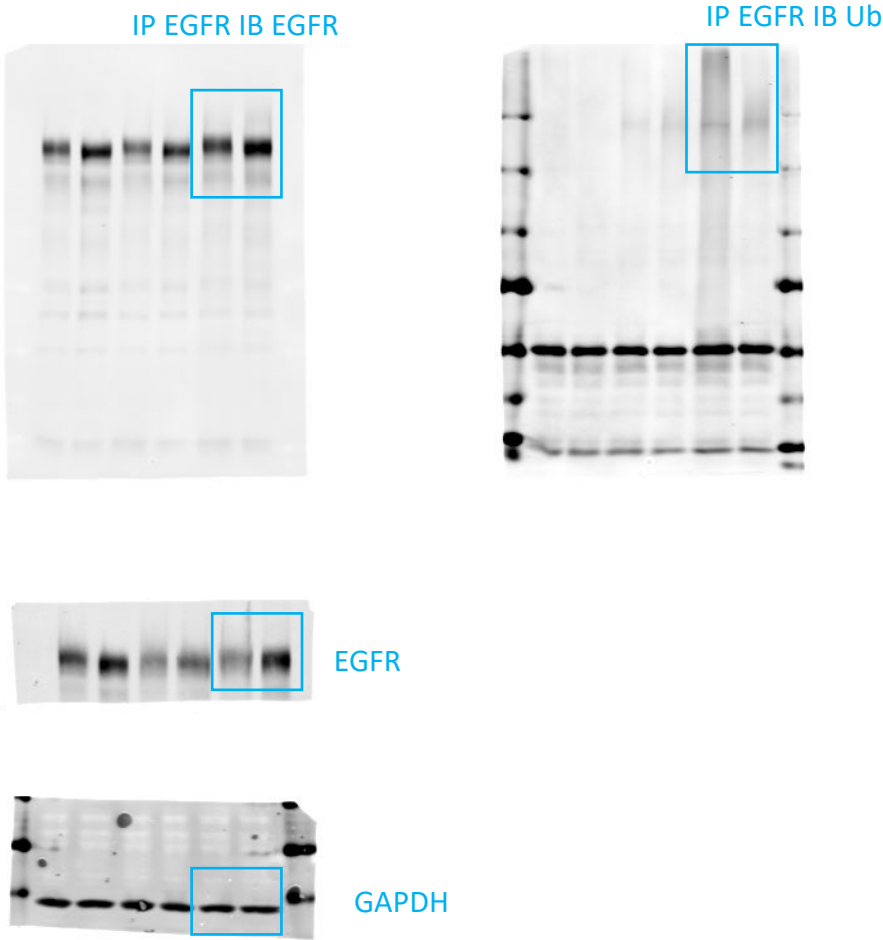

Supplement: Figure 4—source data 1. [file elife-95639-fig4-data1.zip › Figure 4B.pdf]

Figure 4C

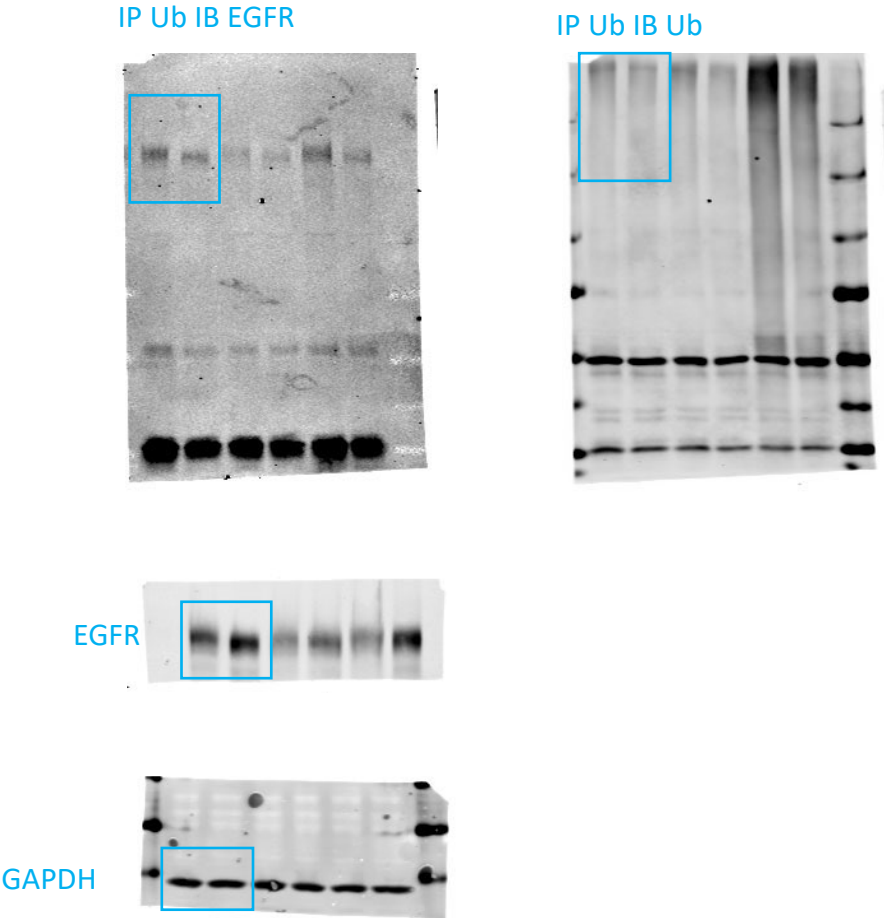

Supplement: Figure 4—source data 1. [file elife-95639-fig4-data1.zip › Figure 4C.pdf]

Figure 4D

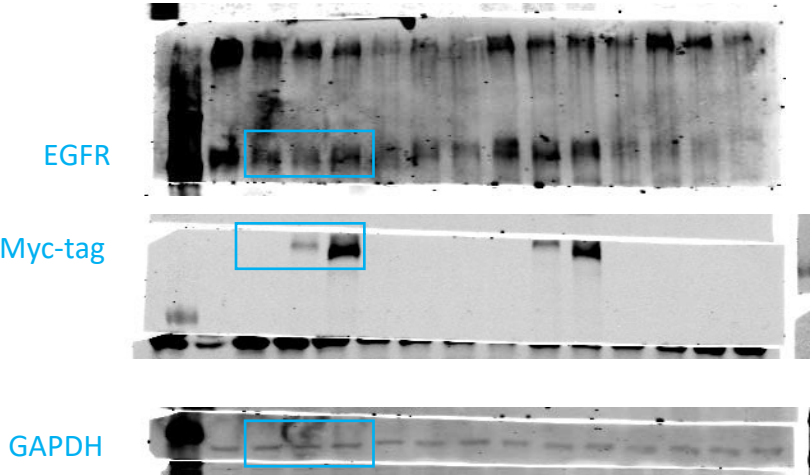

Supplement: Figure 4—source data 1. [file elife-95639-fig4-data1.zip › Figure 4D.pdf]

Figure 4E

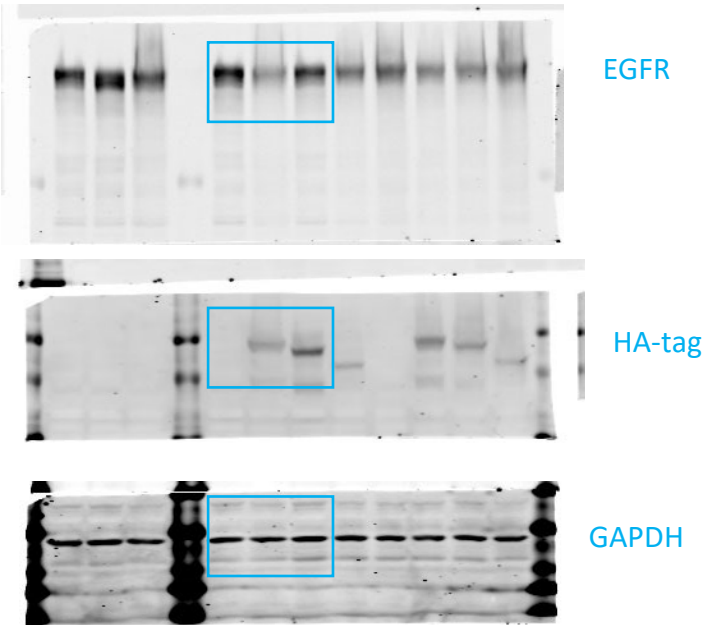

Supplement: Figure 4—source data 1. [file elife-95639-fig4-data1.zip › Figure 4E.pdf]

Figure 4F

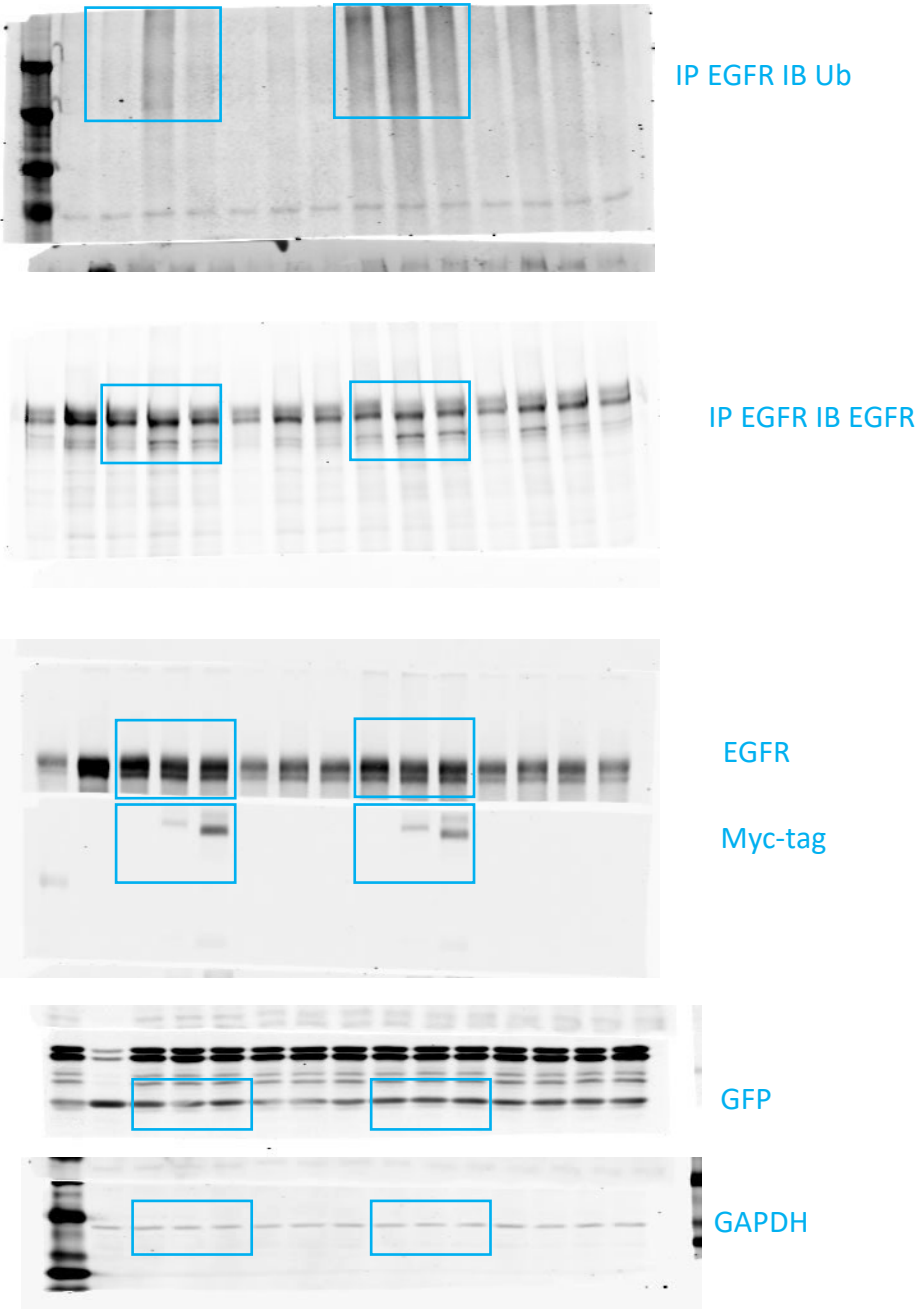

Supplement: Figure 4—source data 1. [file elife-95639-fig4-data1.zip › Figure 4F.pdf]

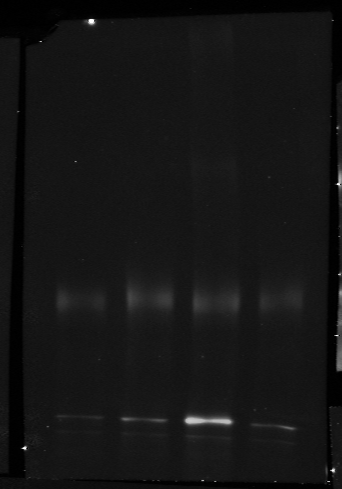

Supplement: Figure 4—source data 2. [file elife-95639-fig4-data2.zip › Fig4A-Input_Myc-tag_800.tif]

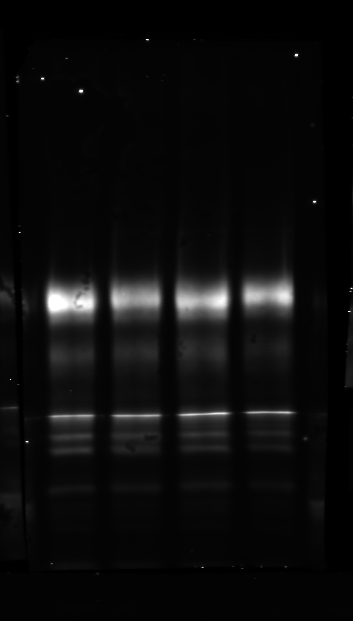

Supplement: Figure 4—source data 2. [file elife-95639-fig4-data2.zip › Fig4A-IP_EGFR_800.tif]

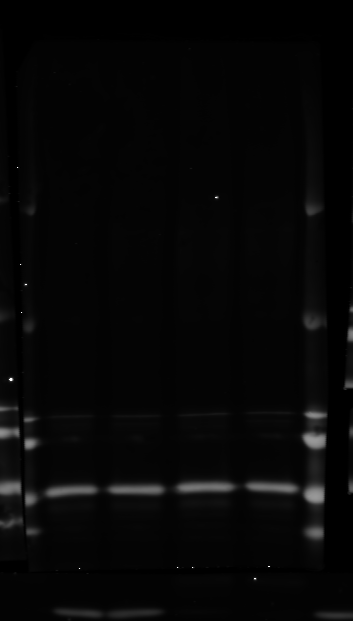

Supplement: Figure 4—source data 2. [file elife-95639-fig4-data2.zip › Fig4A-IP_UB_700.tif]

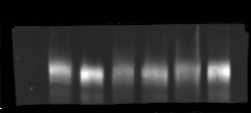

Supplement: Figure 4—source data 2. [file elife-95639-fig4-data2.zip › Fig4B-Input_EGFR_800.tif]

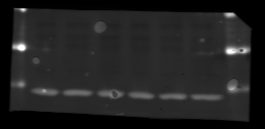

Supplement: Figure 4—source data 2. [file elife-95639-fig4-data2.zip › Fig4B-Input_GAPDH_700.tif]

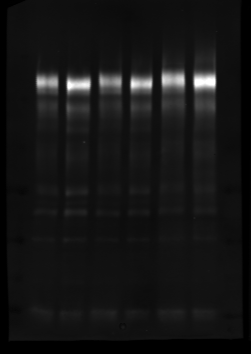

Supplement: Figure 4—source data 2. [file elife-95639-fig4-data2.zip › Fig4B-IP_EGFR_800.tif]

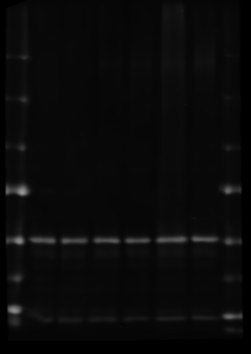

Supplement: Figure 4—source data 2. [file elife-95639-fig4-data2.zip › Fig4B-IP_UB_700.tif]

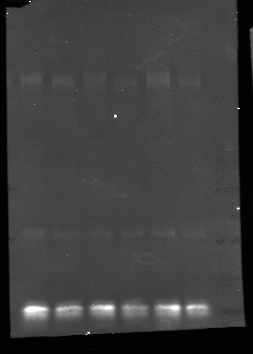

Supplement: Figure 4—source data 2. [file elife-95639-fig4-data2.zip › Fig4C-IP_EGFR_800.tif]

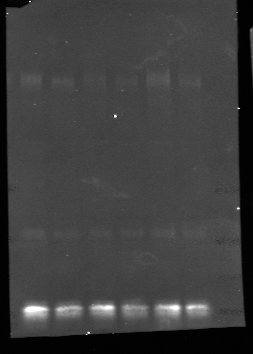

Supplement: Figure 4—source data 2. [file elife-95639-fig4-data2.zip › Fig4C-IP_UB_700.tif]

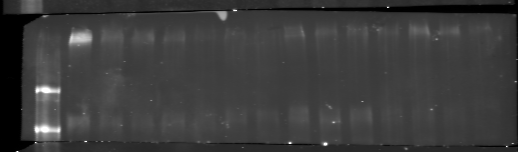

Supplement: Figure 4—source data 2. [file elife-95639-fig4-data2.zip › Fig4D-EGFR_700.tif]

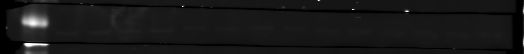

Supplement: Figure 4—source data 2. [file elife-95639-fig4-data2.zip › Fig4D-GAPDH_700.tif]

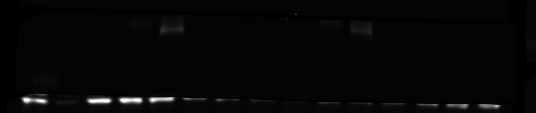

Supplement: Figure 4—source data 2. [file elife-95639-fig4-data2.zip › Fig4D-Myc_800.tif]

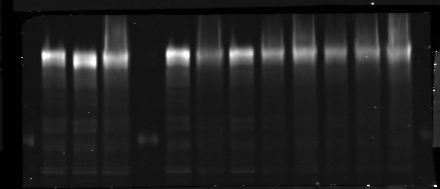

Supplement: Figure 4—source data 2. [file elife-95639-fig4-data2.zip › Fig4E-EGFR_800.tif]

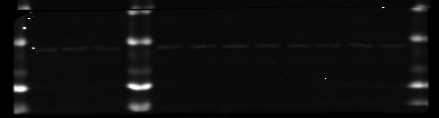

Supplement: Figure 4—source data 2. [file elife-95639-fig4-data2.zip › Fig4E-GAPDH_700.tif]

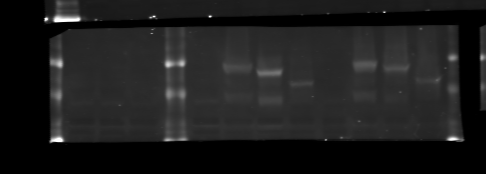

Supplement: Figure 4—source data 2. [file elife-95639-fig4-data2.zip › Fig4E-HA_700.tif]

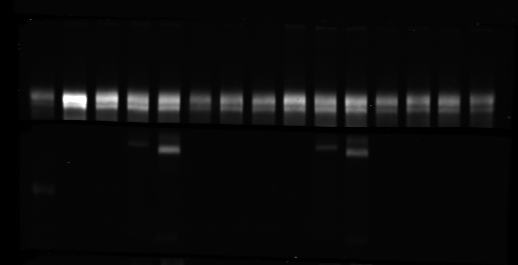

Supplement: Figure 4—source data 2. [file elife-95639-fig4-data2.zip › Fig4F-Input_EGFR_Myc_800.tif]

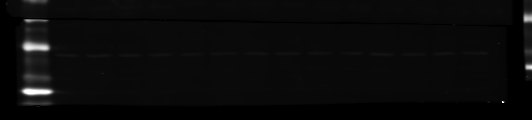

Supplement: Figure 4—source data 2. [file elife-95639-fig4-data2.zip › Fig4F-Input_GAPDH_700.tif]

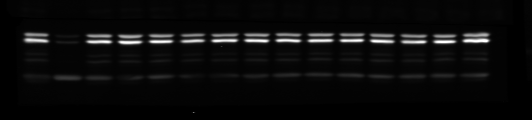

Supplement: Figure 4—source data 2. [file elife-95639-fig4-data2.zip › Fig4F-Input_GFP_800.tif]

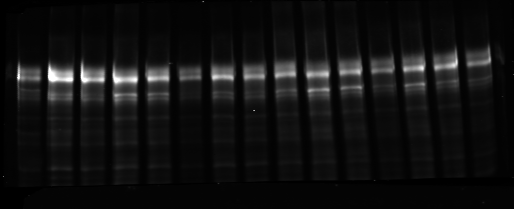

Supplement: Figure 4—source data 2. [file elife-95639-fig4-data2.zip › Fig4F-IP_EGFR_800.tif]

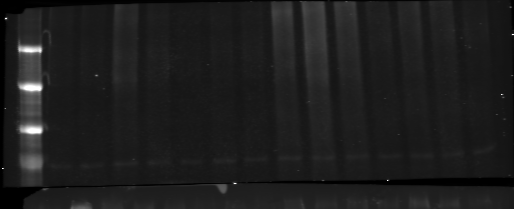

Supplement: Figure 4—source data 2. [file elife-95639-fig4-data2.zip › Fig4F-IP_UB_700.tif]

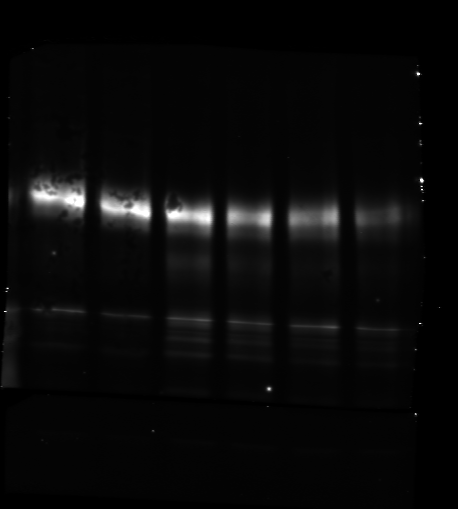

Supplement: Figure 4—source data 2. [file elife-95639-fig4-data2.zip › Fig4A-Input_EGFR_800.tif]

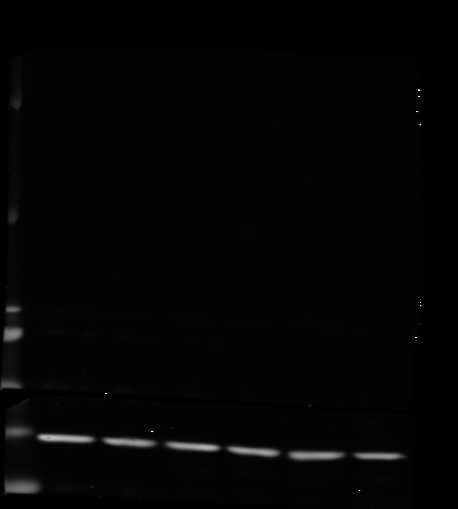

Supplement: Figure 4—source data 2. [file elife-95639-fig4-data2.zip › Fig4A-Input_GAPDH_700.tif]

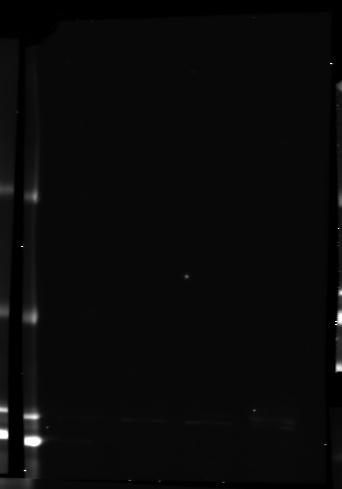

Supplement: Figure 4—source data 2. [file elife-95639-fig4-data2.zip › Fig4A-Input_HA-tag_700.tif]

Figure 4 - Figure supplement 1A

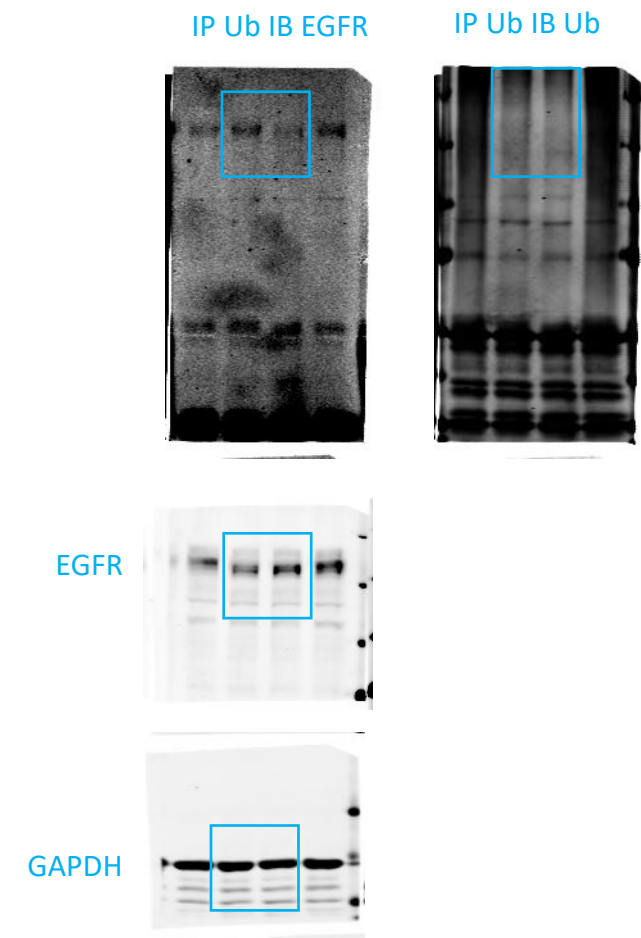

Supplement: Figure 4—figure supplement 1—source data 2. [file elife-95639-fig4-figsupp1-data2.zip › Figure 4 - Figure supplement 1A.pdf]

Figure 4 - Figure supplement 1B

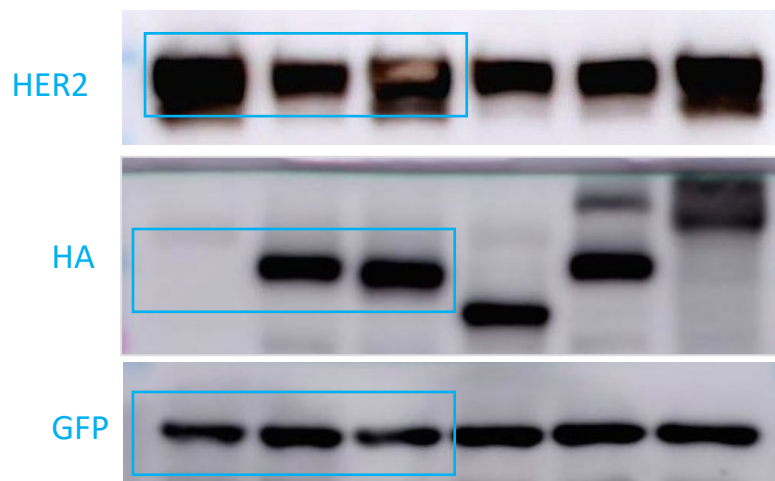

Supplement: Figure 4—figure supplement 1—source data 2. [file elife-95639-fig4-figsupp1-data2.zip › Figure 4 - Figure supplement 1B.pdf]

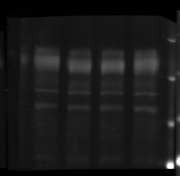

Supplement: Figure 4—figure supplement 1—source data 3. [file elife-95639-fig4-figsupp1-data3.zip › Fig4S1A - Input_EGFR_700.tif]

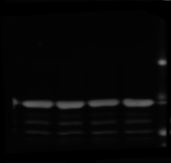

Supplement: Figure 4—figure supplement 1—source data 3. [file elife-95639-fig4-figsupp1-data3.zip › Fig4S1A - Input_GAPDH_700.tif]

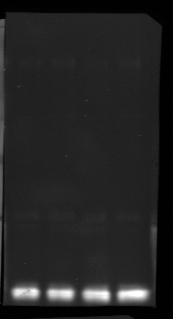

Supplement: Figure 4—figure supplement 1—source data 3. [file elife-95639-fig4-figsupp1-data3.zip › Fig4S1A - IP_EGFR_800.tif]

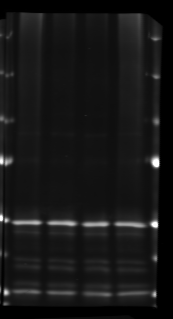

Supplement: Figure 4—figure supplement 1—source data 3. [file elife-95639-fig4-figsupp1-data3.zip › Fig4S1A - IP_UB_700.tif]

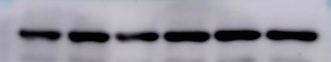

Supplement: Figure 4—figure supplement 1—source data 3. [file elife-95639-fig4-figsupp1-data3.zip › Fig4S1B - GFP.tif]

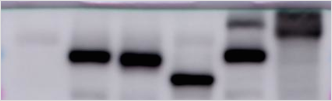

Supplement: Figure 4—figure supplement 1—source data 3. [file elife-95639-fig4-figsupp1-data3.zip › Fig4S1B - HA.tif]

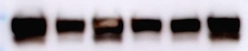

Supplement: Figure 4—figure supplement 1—source data 3. [file elife-95639-fig4-figsupp1-data3.zip › Fig4S1B - HER2.tif]

Figure 5A

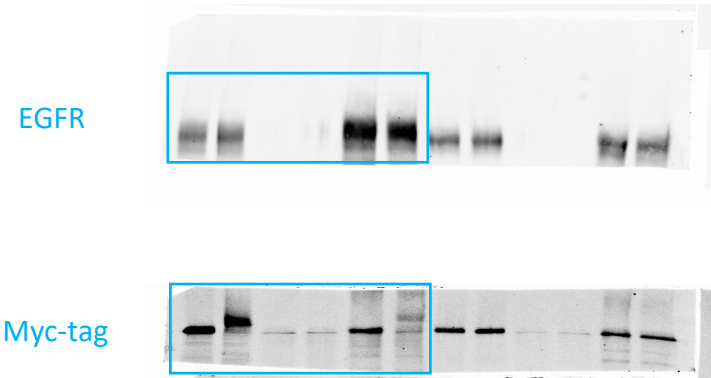

Supplement: Figure 5—source data 1. [file elife-95639-fig5-data1.zip › Figure 5A.pdf]

Figure 5E

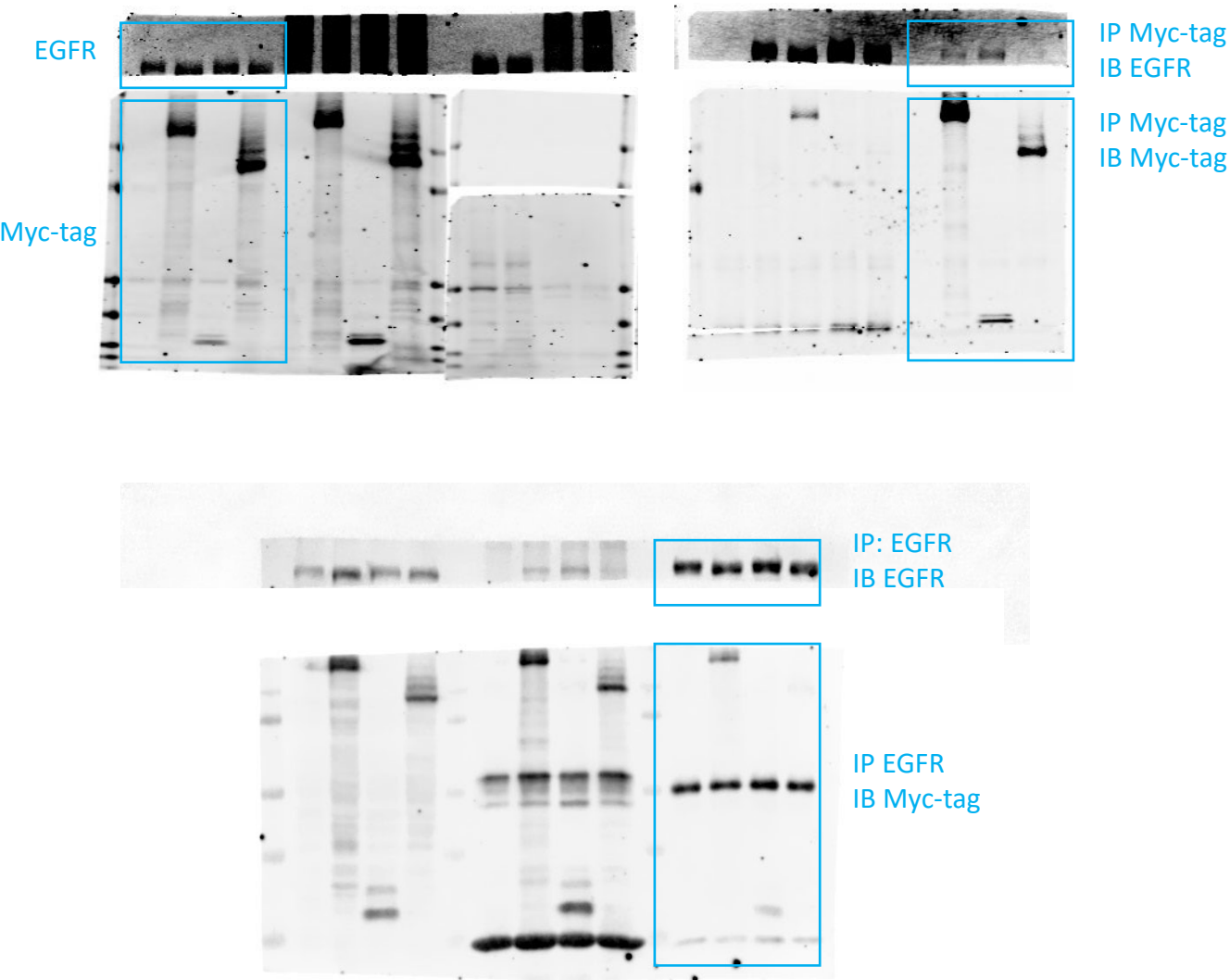

Supplement: Figure 5—source data 1. [file elife-95639-fig5-data1.zip › Figure 5E.pdf]

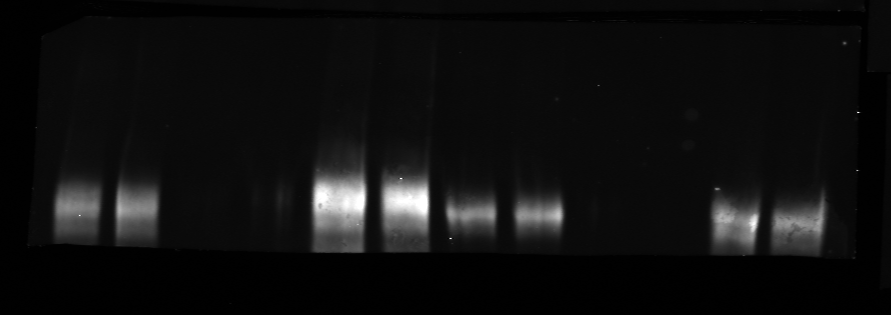

Supplement: Figure 5—source data 2. [file elife-95639-fig5-data2.zip › Fig5A-EGFR_800.tif]

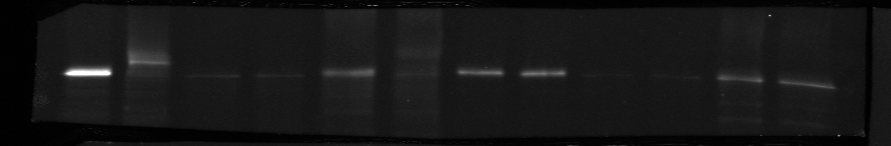

Supplement: Figure 5—source data 2. [file elife-95639-fig5-data2.zip › Fig5A-Myc_800.tif]

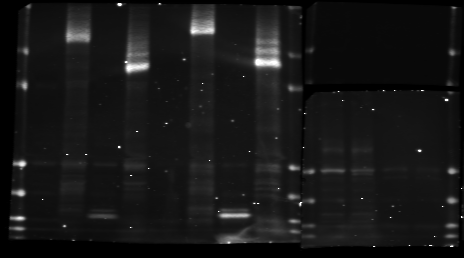

Supplement: Figure 5—source data 2. [file elife-95639-fig5-data2.zip › Fig5E-.tif]

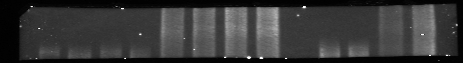

Supplement: Figure 5—source data 2. [file elife-95639-fig5-data2.zip › Fig5E-Input_EGFR_800.tif]
