# Supplementary figures and images for: Loss of ZNRF3/RNF43 unleashes EGFR in cancer (part 2 of 2)
Source: eLife. 2026 Apr 10;13:RP95639. doi: 10.7554/eLife.95639 (PMC13068435; doi:10.7554/eLife.95639)

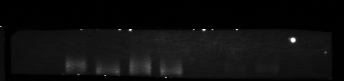

Supplement: Figure 5—source data 2. [file elife-95639-fig5-data2.zip › Fig5E-IP-Myc-EGFR-800.tif]

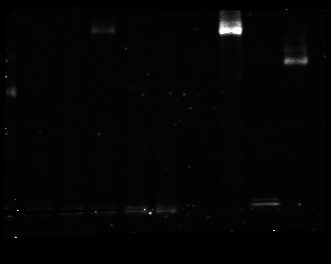

Supplement: Figure 5—source data 2. [file elife-95639-fig5-data2.zip › Fig5E-IP-Myc-Myc-800.tif]

Figure 5 - Figure supplement 1C

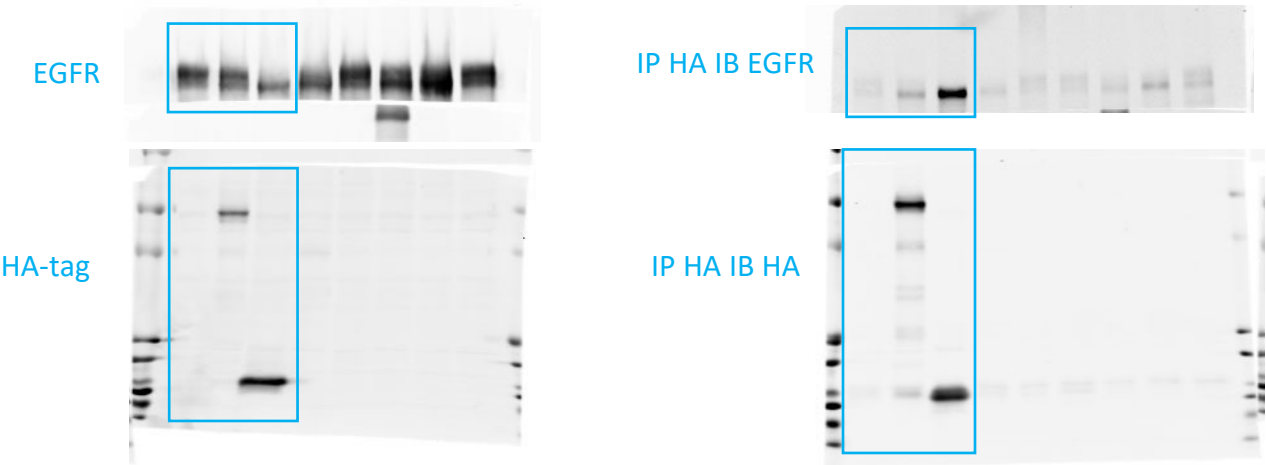

Supplement: Figure 5—figure supplement 1—source data 1. [file elife-95639-fig5-figsupp1-data1.zip › Figure_5_-_Figure_supplement_1C.pdf]

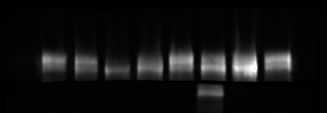

Supplement: Figure 5—figure supplement 1—source data 2. [file elife-95639-fig5-figsupp1-data2.zip › Fig5S1C - Input_EGFR_800.tif]

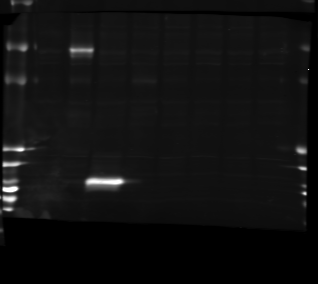

Supplement: Figure 5—figure supplement 1—source data 2. [file elife-95639-fig5-figsupp1-data2.zip › Fig5S1C - Input_HA_700.tif]

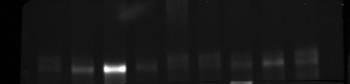

Supplement: Figure 5—figure supplement 1—source data 2. [file elife-95639-fig5-figsupp1-data2.zip › Fig5S1C - IP_EGFR_800.tif]

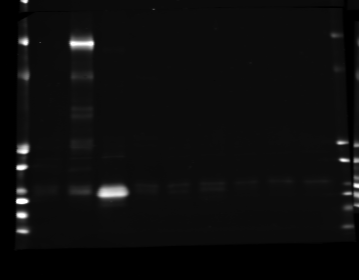

Supplement: Figure 5—figure supplement 1—source data 2. [file elife-95639-fig5-figsupp1-data2.zip › Fig5S1C - IP_HA_700.tif]

Figure 5 - Figure supplement 2B

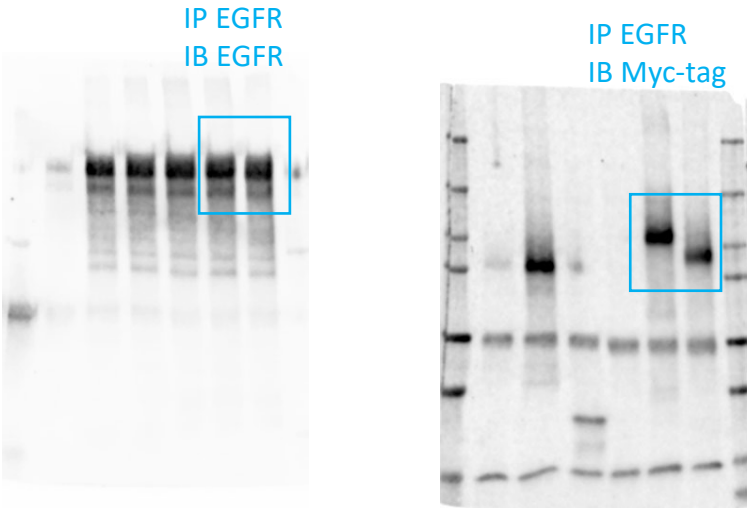

Supplement: Figure 5—figure supplement 2—source data 2. [file elife-95639-fig5-figsupp2-data2.zip › Figure 5 - Figure supplement 2B.pdf]

Figure 5 - Figure supplement 2C

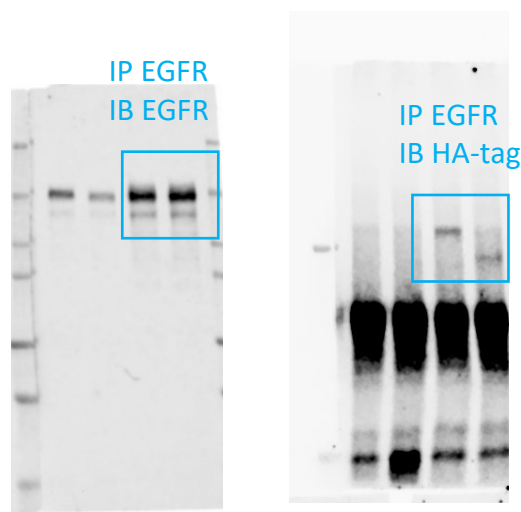

Supplement: Figure 5—figure supplement 2—source data 2. [file elife-95639-fig5-figsupp2-data2.zip › Figure 5 - Figure supplement 2C.pdf]

Figure 6J

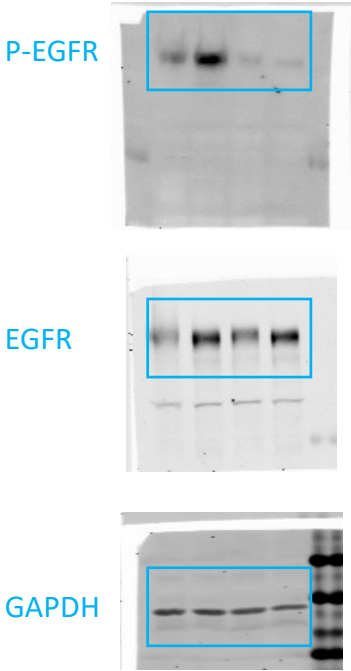

Supplement: Figure 6—source data 2. [file elife-95639-fig6-data2.zip › Figure 6J.pdf]

Figure 6F

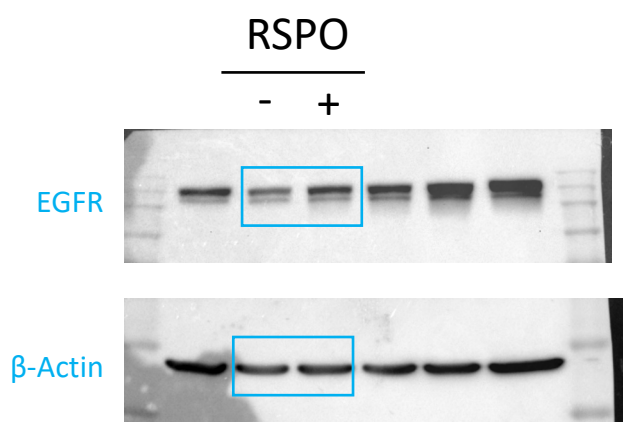

*Apc*<sup>min</sup> mouse intestinal tumor organoid

Supplement: Figure 6—source data 2. [file elife-95639-fig6-data2.zip › Figure 6F.pdf]

Figure 6l

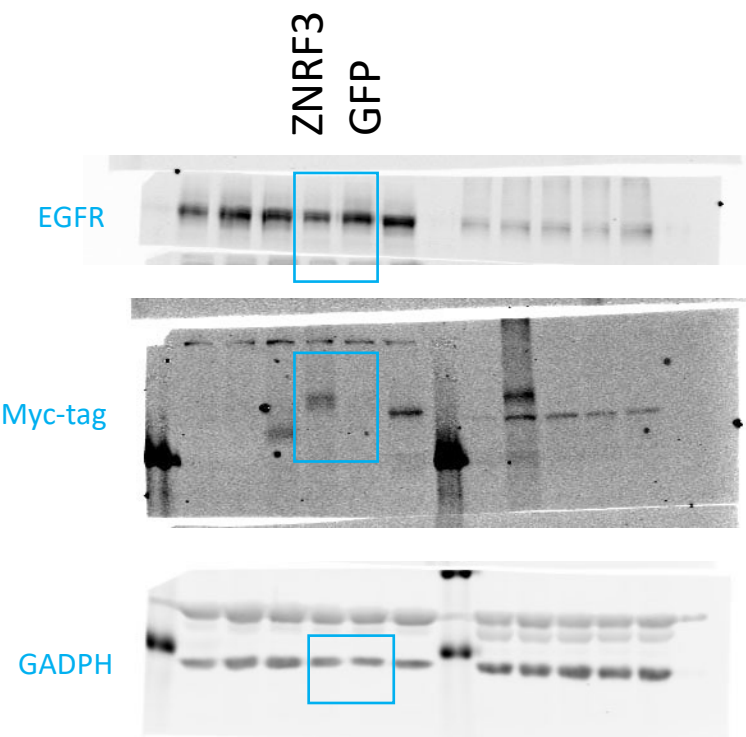

Supplement: Figure 6—source data 2. [file elife-95639-fig6-data2.zip › Figure 6I.pdf]

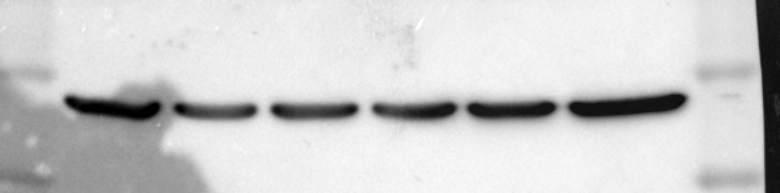

Supplement: Figure 6—source data 3. [file elife-95639-fig6-data3.zip › Fig6F - bactin.tif]

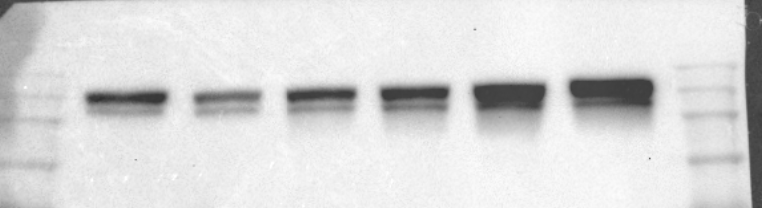

Supplement: Figure 6—source data 3. [file elife-95639-fig6-data3.zip › Fig6F - EGFR.tif]

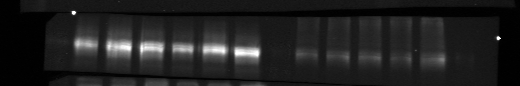

Supplement: Figure 6—source data 3. [file elife-95639-fig6-data3.zip › Fig6I-EGFR-800.tif]

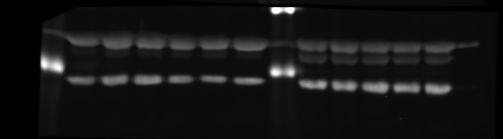

Supplement: Figure 6—source data 3. [file elife-95639-fig6-data3.zip › Fig6I-GAPDH-700.tif]

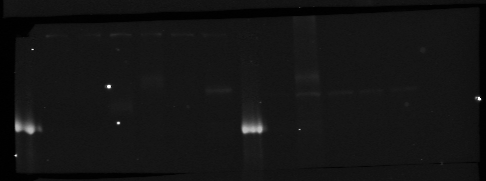

Supplement: Figure 6—source data 3. [file elife-95639-fig6-data3.zip › Fig6I-Myc-800.tif]

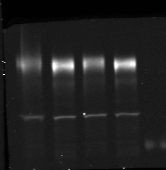

Supplement: Figure 6—source data 3. [file elife-95639-fig6-data3.zip › Fig6J-EGFR-800.tif]

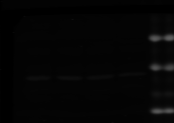

Supplement: Figure 6—source data 3. [file elife-95639-fig6-data3.zip › Fig6J-GAPDH-700.tif]

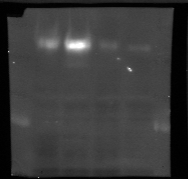

Supplement: Figure 6—source data 3. [file elife-95639-fig6-data3.zip › Fig6J-pEGFR-800.tif]

Figure 7B

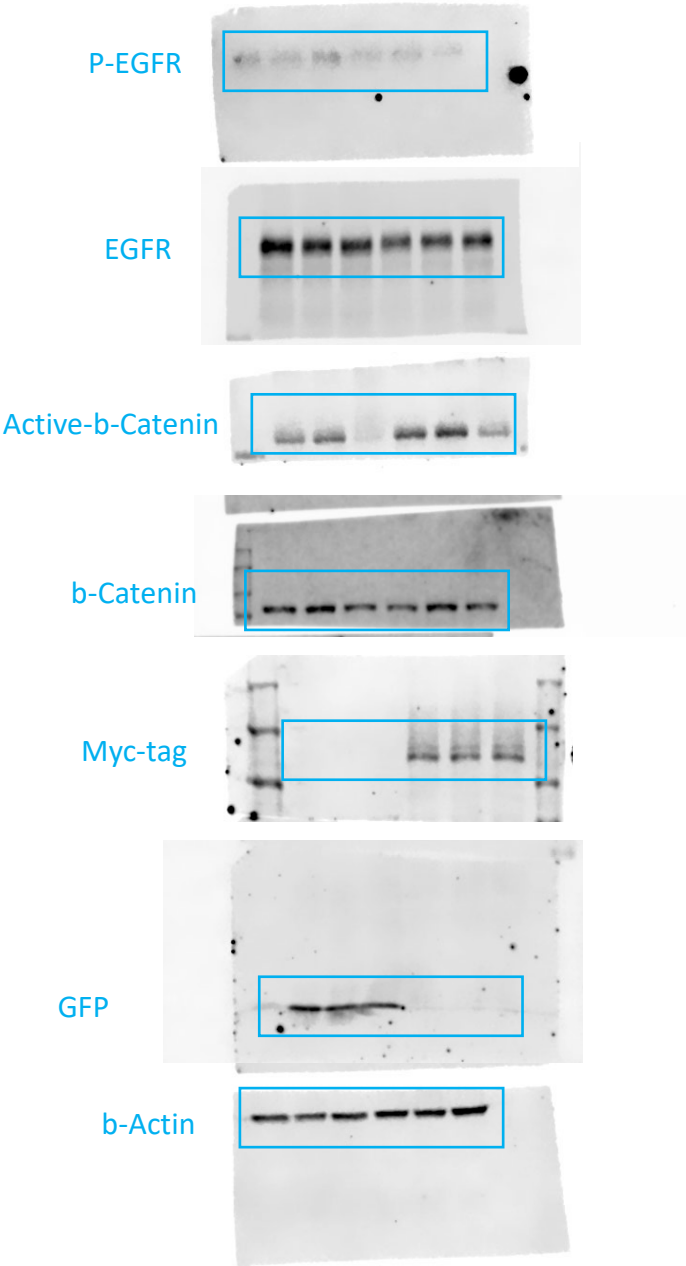

Supplement: Figure 7—source data 2. [file elife-95639-fig7-data2.zip › Figure_7_source_data_2--Figure_7B.pdf]
